# Assessment of oral toxicity of *Moringa oleifera* Lam aqueous extract and its effect on gout induced in a murine model

**DOI:** 10.14202/vetworld.2024.1449-1458

**Published:** 2024-07-07

**Authors:** Miriam Palomino-Pacheco, Juan Pedro Rojas-Armas, José Manuel Ortiz-Sánchez, Jorge Luis Arroyo-Acevedo, Hugo Jesús Justil-Guerrero, Jaime Teodocio Martínez-Heredia

**Affiliations:** 1 Section of Biochemistry, Faculty of Medicine, Universidad Nacional Mayor de San Marcos, Lima, Peru; 2 Section of Pharmacology, Faculty of Medicine, Universidad Nacional Mayor de San Marcos, Lima, Peru; 3 Section of Physiology, Faculty of Medicine, Universidad Nacional Mayor de San Marcos, Lima, Peru

**Keywords:** extract, gout, *Moringa oleifera*, murine, toxicity

## Abstract

**Background and Aim:**

Although widely employed in traditional remedies globally, the safety and efficacy of *Moringa oleifera* remain inadequately documented through scientific research. This study evaluated the oral toxicity of *M. oleifera* leaf aqueous extract (MoAE) and its impact on gout-induced rats.

**Materials and Methods:**

2000 mg/kg was given in a single dose during the acute oral toxicity test, while 100 mg/kg, 250 mg/kg, and 500 mg/kg were given daily for 28 days in the repeated dose toxicity test. 100 mg/kg, 250 mg/kg, and 500 mg/kg MoAE doses were administered during the assessment of its impact on gout caused by monosodium urate. In the hyperuricemia model induced by oxonic acid, serum uric acid levels were assessed and pain response was measured through acetic acid-induced writhing.

**Results:**

In acute oral and 28-day repeated dose tests, no indications of toxicity were detected, while MoAE alleviated ankle joint swelling and reduced serum uric acid concentrations in arthritic rats, causing a significant reduction in acetic acid-induced contortions.

**Conclusion:**

No acute oral toxicity or toxicity in 28-day repeated doses was found for MoAE, while it exhibited antiarthritic, antihyperuricemic, and pain-relieving effects in the murine model.

## Introduction

*Moringa oleifera* Lam *(Moringaceae* family) is native to the sub-Himalayan region of India, Arabia, Asia, Pakistan, and Africa. It can grow up to 10–12 m in height and is now widely distributed around the world [1]. In traditional South Asian medicine, this plant’s various components have been employed to treat numerous diseases [2].

*M. oleifera* seeds are known for their pain-relief properties [3], but research primarily focuses on leaves. Nevertheless, aqueous extracts reveal high antioxidant activity and antiproliferative effects against breast cancer cells [4], activity against *Streptococcus mutans* and *Proteus vulgaris* [5], anti-inflammatory effects [6], potential to inhibit the initial stages of inflammation and contribute significantly to modulation of chronic inflammation [7], and alcoholic extracts display anti-arthritic effects [8].

Gout occurs due to the presence of monosodium urate (MSU) crystals in joints and tissue, capable of inciting inflammatory responses and severe discomfort during attacks [9]. Peripheral joint synovitis attacks with acute pain and self-resolution often serve as the early manifestation, but lesions, deformities, and chronic pain due to subcutaneous tophi accumulation may ensue later [10]. The global prevalence of gout ranges between 1% and 4%, and new cases number from 0.1% to 0.3%. The frequency of the condition is 3–10 times higher in men than in women. In addition, the likelihood of experiencing gout increases with each successive decade of life [11]. Treatment of gout aims to reduce uricemia (allopurinol) and decrease joint inflammation with non-steroidal anti-inflammatory drugs, corticosteroids, interleukin (IL)-1 inhibitor, and old colchicine [12]. Due to the significant adverse effects of these drugs, there is a need to explore novel, safe, and effective alternatives.

Drug development requires toxicity assessment during its early stages before clinical trials. This is done to gather pertinent data for future decisions and prevent potential adverse effects on the organism, ensuring safe use of these drugs in humans [13]. Although herbal treatments are commonly perceived as safe and beneficial because of their natural origin, this is not always true because plants produce some metabolites with toxic properties that they use to defend themselves against threatening species [14]. Safety and efficacy studies are essential to promote the integration of traditional and modern medicine [15].

Significant research is being conducted to prove the therapeutic properties and safety of medicinal plants. Due to its various applications in traditional medicine, *M. oleifera* is referred to as the “miracle medicinal plant” [16], and numerous scientific investigations have been undertaken; however, it has recently been suggested that additional evaluation is required for the pharmacological and toxicological aspects of moringa extracts [17].

This study aimed to evaluate both the acute and repeated dose toxicity of *M. oleifera* leaf aqueous extract (MoAE), as well as its potential impact on gout using a murine model, based on prior research on its anti-inflammatory properties.

## Materials and Methods

### Ethical approval

This study was approved by the Ethics Committee of the Faculty of Pharmacy and Biochemistry of the Universidad Nacional Mayor de San Marcos (Approval Certificate N° 007- CE-UDI- FFB-2020).

### Study period and location

This study was conducted from March 2022 to December 2022 at the Laboratory of Experimental Pharmacology, Faculty of Medicine, Universidad Nacional Mayor de San Marcos, Lima, Peru.

### Botanical material

*M. oleifera* leaves were collected from the Trujillo Province in Peru. A specimen of the plant was brought to the herbarium in San Marcos within the UNMSM Natural History Museum for taxonomic categorization (Reference Number 249-USM-2021).

### Biological material

This study employed Holtzman strain albino rats acquired from the Instituto Nacional de Salud, Peru. Before the study’s commencement, the animals were acclimatized in the laboratory for a week. The animals lived in a controlled habitat with a 12-h light cycle and a temperature of 22 ± 3°C. They were given commercial rat food and unlimited water.

### Extract preparation

The MoAE was prepared by washing, drying, grinding, boiling, and filtering: The leaves were first washed with tap water, followed by drying at 40°C until completely dry. The dried leaves were ground into a powder using an electric blade mill. This powder was then added to a vessel containing boiling water and stirred for a quarter hour. The resulting mixture was filtered using a vacuum filtration apparatus. The water was removed through a rotary evaporator at 40°C. The MoAE residue, kept at 4°C, was used as needed.

### Preliminary phytochemical screening [18]

The secondary metabolites of MoAE were identified as phenolic compounds, flavonoids, tannins, naphthoquinones, anthraquinones, anthrones, alkaloids, cardiotonic heterosides, saponins, glycosides, and terpenes, using common reagents.

### Evaluation of the antioxidant capacity

*Total antioxidant capacity assay* [19]. Extract concentrations were prepared from 10 to 320 μg/mL; afterward, 2.5 mL of each of these concentrations was combined with 2.5 mL of 0.2 M sodium phosphate buffer (pH 6.6) and 2.5 mL of 1% potassium ferricyanide. They were placed in an oven and subjected to a temperature of 50°C for 20 min. Following the elapsed time, 2.5 mL of trichloroacetic acid was introduced at a concentration of 10% (w/v), mixed, and centrifuged at 1000× *g* for 8 min. Subsequently, 5 mL of the resulting supernatant was combined with 5 mL of distilled water and 1 mL of 0.1% ferric chloride. The spectrophotometric measurement of absorbance was conducted at a wavelength of 700 nm. The experiment was performed 3 times, and the results were presented as average values accompanied by standard deviations. The mean effective concentration (EC_50_) was determined by analyzing the graph correlating the absorbance at 700 nm with the extract concentration. The standard antioxidant used was ascorbic acid.

### Toxicity assessment

#### Investigations of acute oral toxicity [20]

In accordance with the Organisation for Economic Cooperation and Development (OECD) Method 423, three 160 ± 10 g female rats were given a single 2000 mg/kg oral dose of MoAE after an overnight fast. Close monitoring continued for the first 30 min, the next 4 h, and the remaining 14 days. Animal mortality and signs of toxicity were closely monitored. After the experiment’s completion, the animals were euthanized with ethyl ether, followed by necropsy and extensive anatomical analysis using macroscopic and microscopic methods.

#### Oral toxicity study with repeated doses over a period of 28 days [21]

Twenty female rats of 160 ± 10 g body weight and 20 male rats of 170 ± 10 g body weight were used and randomly assigned to four groups (n = 10: 5 females and 5 males). Rats in the control group (Group I) were given vehicle alone for 28 days, while Groups II, III, and IV received MoAE daily at oral doses of 100, 250, and 500 mg/kg body weight, respectively. Animals were closely monitored daily for 28 days to detect toxicity or mortality. Twenty-nine-day post-anesthesia, blood samples were gathered through intracardiac puncture for hematological and biochemical analysis. An intraperitoneal injection of a sodium pentobarbital overdose (80 mg/kg) was administered for euthanasia. Organ specimens underwent histopathological analysis after preservation in 10% formalin solution.

#### Recording of the body mass

The individual weights of the rats were documented once a week throughout the research period.

#### Assessment of the biochemical indicators

A semiautomatic analyzer (URIT-810, URIT Medical Electronic Co., Guangxi, China) was employed for biochemical tests. According to the manufacturer’s protocols, biochemical indicators were measured. These thirteen components were quantified: alkaline phosphatase, aspartate aminotransferase (AST), total protein, alanine aminotransferase (ALT), bilirubin, cholesterol, glucose, creatinine, triglycerides, high-density lipoprotein, low-density lipoproteins (LDL), urea, and total albumin.

#### Hematological parameters

A complete blood count was conducted, measuring erythrocyte count, leukocyte count, differential hematocrit, hemoglobin concentration, and platelet count. A Genrui KT-6400 automatic hematology analyzer (Genrui Biotech Inc., Shenzhen, China) was employed.

#### Histopathological analysis

The animal organs were preserved in a 10% formalin solution. They were preserved by dehydrating and encasing in paraffin for 3 days. 5 μm sections were prepared from the tissue using a microtome and stained with hematoxylin/eosin. The histopathological examination was conducted through light microscopy (Leica Microsystems, Wetzlar, Germany) at 400× magnification.

### Assessment of the effect on gout

#### Effect on MSU crystal-induced arthritis in rats [22]

Thirty-six male albino rats weighing 180–220 g distributed in six groups were used: Group I, normal control; Group II, induced; Group III, colchicine 0.30 mg/kg/day (positive control); and Groups IV, V, and VI, each administered MoAE at doses of 100, 250, and 500 mg/kg/day, respectively. On the 6^th^ day, every rat, excluding those in the normal control group, received an injection of 0.1 mL MSU crystal suspension at a concentration of 30 mg/mL into the synovial space of the joint of the right ankle in the hind paw 30 min before daily administration of the treatment. Furthermore, the control group designated as normal received 0.1 mL of 0.9% saline solution through the same procedural manipulation. On the 9^th^ day, the circumference of the right ankle was measured using a vernier caliper at intervals of 0, 4, 12, and 24 h after the last treatment. The diameters of the ankle joint in each rat were measured at three random positions and their averages were used to determine the circumference. The computations were based on the following formula.

Percent swelling (%) = (Ct–C0)/C0

Here, Ct signifies the circumference at various time points, whereas C0 signifies the circumference at the starting point (0 h).

The animals were sacrificed using an intraperitoneal injection of 80 mg/kg sodium pentobarbital. Ankle joints from gouty arthritis patients were decalcified using 10% ethylenediaminetetraacetic acid before being preserved in 10% formaldehyde. 5 μm thin sections were cut from paraffin-embedded joints treated with various alcohol/xylene mixtures. After staining with hematoxylin and eosin, they were examined under light microscopy.

#### Effect on oxonic acid-induced hyperuricemia [23]

Thirty-six male albino rats weighing 150 ± 20 g were distributed in six groups (n = 6): Group 1 received distilled water, served as normal control; Group 2 received intraperitoneally saline and oxonic acid (250 mg/kg), served as induced control (oxonate group); Group 3 received allopurinol (50 mg/kg) and oxonic acid, served as positive control; and Groups 4, 5, and 6 received MoAE in doses of 100, 250, and 500 mg/kg, respectively, and oxonic acid. Each group received intragastric treatment once daily for 7 days. On the 1^st^, 3^rd^, and 7^th^ days of the experiment, intraperitoneal injections of oxonic acid (250 mg/kg) were administered 1 h before the animals were treated with the extract or allopurinol, excluding the normal control group. On the 7^th^ day of the experiment, 2 h after the last treatment, all groups underwent anesthesia and the animals were sacrificed to collect blood samples. Blood was allowed to coagulate at room temperature (22°C) for approximately 1 h, followed by centrifugation at 3000× *g* for 5 min to obtain serum. Uric acid levels in serum were evaluated following the manufacturer’s guidelines using a semi-automatic biochemical analyzer (URIT-810, URIT Medical Electronic Co.).

#### Evaluation of analgesic activity [24]

The acetic acid-induced writhing method was used. The mice were divided into five groups, each comprising six individuals. Briefly, abdominal contractions were triggered by injecting 1% acetic acid (10 mL/kg, ip) after a 30-min interval after vehicle administration (Group 1), tramadol 20 mg/kg (Group 2), and MoAE (Groups 3, 4, and 5) at doses of 100, 250, and 500 mg/kg, respectively. The count of abdominal contractions was observed and documented in 15 min. The percentage inhibition of writhing was determined using the following formula:

% inhibition = (Average number of writhes [control] − Average number of writhes [test]/Average number of writhes [control]) × 100

### Statistical analysis

Data are presented as mean ± standard deviation and subjected to one-way analysis of variance, followed by a *post hoc* Tukey multiple comparison test using Statistical Package for the Social Sciences software version 20 for analysis (IBM Corp., NY, USA). Statistical significance was attributed to values with p < 0.05.

## Results

### Chemical analysis and antioxidant capacity

Phytochemical screening of MoAE showed that the most abundant secondary metabolites were phenolic compounds, flavonoids, and tannins (Table-1). The Fe^3+^ reducing power assay revealed an antioxidant capacity of 7.91 ± 0.10 μg ascorbic acid equivalent per milligram.

**Table-1:** Secondary metabolites of MoAE.

Reagent	Secondary metabolite	Result
Ferric chloride	Phenolic compounds	(+++)
Shinoda	Flavonoids	(+++)
Gelatin	Tannins	(++)
Bortranger	Naphthoquinones, Anthraquinones, Anthrones	(+)
Lieberman- Bouchardat	Triterpenoids	(+)
Dragendorff	Alkaloids	(-)
Wagner	Alkaloids	(-)
Bertrand	Alkaloids	(-)
Popoff	Alkaloids	(-)
Mayer	Alkaloids	(-)
Baljet	Cardiotonic heterosides and sesquiterpenlactones	(++)
Afrosimetric indices	Saponins	(+)
Vanillin/H_2_SO_4_	Glycosides	(+)
Salkowsky	Terpenoids	(-)

High evidence (+++), medium evidence (++), low evidence (+), no evidence (-), MoAE=*M. oleifera* leaf aqueous extract

### Acute toxicity

In an oral toxicity evaluation carried out following OECD guideline 423, using a maximum dose of 2000 mg/kg body weight, no indications of toxicity were observed in rats within the initial 4 h after MoAE administration. In addition, there were no signs of toxicity throughout the 14-day monitoring period. According to the global harmonized system of classification, the substance falls into category 5 or is not classified (non-toxic), with an LD_50_ cutoff point of 5000 mg/kg body weight. Macroscopic or microscopic alterations were not observed in the organs.

### Repeated dose toxicity

In the 28-day repeated-dose oral toxicity test, no rats died during the study or showed signs of toxicity. There was no significant change in body weight gain for female and male rats subjected to MoAE treatment over a 28-day period compared with the control group (Table-2).

**Table-2:** Body weight growh in rats (grams) during a 28-day regimen of repeated MoAE doses.

	Week 0	Week 1	Week 2	Week 3	Week 4
Males					
Control	209.80 ± 3.03	248.20 ± 7.05	271.40 ± 7.12	294.80 ± 8.04	315.40 ± 16.44
Moringa 100	205.20 ± 6.06	241.00 ± 13.98	260.40 ± 11.84	280.20 ± 12.81	299.20 ± 9.81
Moringa 250	208.00 ± 8.86	240.00 ± 15.49	257.20 ± 11.78	275.00 ± 16.32	293.00 ± 8.34
Moringa 500	208.20 ± 6.98	245.00 ± 13.87	267.80 ± 21.49	291.20 ± 23.08	304.80 ± 17.48
Females					
Control	169.00 ± 7.87	202.40 ± 10.50	218.20 ± 10.64	230.00 ± 13.34	240.40 ± 12.28
Moringa 100	166.20 ± 14.41	185.80 ± 15.29	203.40 ± 10.74	217.00 ± 10.37	227.60 ± 7.30
Moringa 250	166.60 ± 12.54	195.80 ± 18.95	210.20 ± 9.42	217.20 ± 12.19	225.40 ± 8.50
Moringa 500	168.20 ± 7.19	199.40 ± 17.85	212.20 ± 13.33	225.00 ± 7.91	237.80 ± 8.93

Data are presented as mean ± standard deviation. There is no notable distinction between the treated groups compared to the control group, MoAE=*M. oleifera* leaf aqueous extract

### Biochemical indicators

Daily administration of *M. oleifera* for 28 days did not result in significant alterations in serum levels of AST, ALT, and alkaline phosphatase in male and female rats compared with the control group. No alteration in lipid profile, glycemia levels, or renal parameters was also observed (Table-3).

**Table-3:** Biochemical parameters in rats during a 28-day regimen of repeated MoAE doses.

Parameters	Control	MoAE 100	MoAE 250	MoAE 500
Males				
AST (IU/L)	181.87 ± 7.95	183.09 ± 11.13	193.95 ± 7.72	194.18 ± 5.97
ALT (IU/L)	75.40 ± 11.95	78.83 ± 6.87	80.38 ± 9.01	82.49 ± 12.60
Alkaline phosphatase (IU/L)	228.63 ± 33.72	238.75 ± 36.73	235.78 ± 33.32	241.78 ± 28.82
Cholesterol (mg/dL)	112.58 ± 9.02	114.28 ± 4.45	116.23 ± 17.91	116.85 ± 11.19
HDL (mg/dL)	32.83 ± 3.04	32.63 ± 2.97	33.35 ± 1.22	34.14 ± 3.54
LDL (mg/dL)	59.89 ± 10.07	62.67 ± 6.97	63.65 ± 14.52	63.13 ± 8.37
Triglycerides (mg/dL)	99.25 ± 7.44	97.33 ± 7.33	96.14 ± 13.77	97.90 ± 11.94
Glucose (mg/dL)	79.78 ± 5.01	79.67 ± 12.59	77.88 ± 5.23	82.77 ± 8.59
Urea (mg/dL)	41.82 ± 2.13	41.45 ± 4.22	47.76 ± 6.66	46.42 ± 3.38
Total protein (g/dL)	7.98 ± 0.30	7.90 ± 0.48	7.95 ± 0.35	7.96 ± 0.36
Albumin (g/dL)	4.40 ± 0.24	4.50 ± 0.34	4.43 ± 0.32	4.48 ± 0.31
Creatinine (mg/dL)	0.45 ± 0.06	0.47 ± 0.08	0.48 ± 0.09	0.53 ± 0.08
Females				
AST (IU/L)	180.10 ± 15.35	188.10 ± 28.42	181.59 ± 13.72	189.29 ± 10.07
ALT (IU/L)	71.85 ± 8.15	76.80 ± 7.98	76.91 ± 14.81	75.07 ± 7.76
Alkaline phosphatase (IU/L)	227.61 ± 22.72	234.74 ± 30.85	239.64 ± 21.13	242.65 ± 16.55
Cholesterol (mg/dL)	115.77 ± 3.95	117.38 ± 9.60	121.30 ± 16.31	126.38 ± 10.01
HDL (mg/dL)	33.03 ± 2.17	32.64 ± 3.02	31.68 ± 2.71	33.37 ± 2.72
LDL (mg/dL)	62.20 ± 5.01	64.72 ± 7.33	69.16 ± 11.71	72.37 ± 9.26
Triglycerides (mg/dL)	102.70 ± 5.53	100.09 ± 5.77	102.29 ± 11.61	103.19 ± 8.05
Glucose (mg/dL)	82.98 ± 6.46	70.86 ± 7.03	70.37 ± 7.06	78.77 ± 14.36
Urea (mg/dL)	46.03 ± 3.42	49.96 ± 3.77	47.78 ± 9.14	49.45 ± 5.84
Total protein (g/dL)	7.99 ± 0.29	7.91 ± 0.46	7.92 ± 0.17	7.88 ± 0.29
Albumin (g/dL)	4.43 ± 0.24	4.49 ± 0.23	4.59 ± 0.38	4.53 ± 0.57
Creatinine (mg/dL)	0.54 ± 0.07	0.55 ± 0.08	0.57 ± 0.09	0.55 ± 0.10

Data are presented as average ± standard deviation, There was no significance versus control. ALT, alanine aminotransferase; AST, aspartate aminotransferase; LDL, low-density lipoprotein; and HDL, high-density lipoprotein, MoAE=*M. oleifera* leaf aqueous extract

### Hematological parameters

The blood cell counts of the animals in the repeated oral dose experiment stayed within normal limits. No alterations were noted in hemoglobin, hematocrit, and leukocyte levels (Table-4).

**Table-4:** Hematological parameters in rats during a 28-day regimen of repeated MoAE doses.

Parameters	Control	MoAE 100	MoAE 250	MoAE 500
Males				
RBC (x10^6^/μL)	7.87 ± 0.41	7.09 ± 1.21	8.01 ± 0.22	8.31 ± 0.25
WBC (x10^3^/μL)	9.73 ± 0.62	9.35 ± 2.07	8.50 ± 1.36	8.03 ± 0.89
Hemoglobin (g/dL)	14.53 ± 2.26	14.33 ± 2.14	14.90 ± 1.87	14.95 ± 2.03
Hematocrit (%)	44.35 ± 1.59	40.60 ± 5.83	44.43 ± 1.12	45.98 ± 1.81
Neutrophils (%)	19.15 ± 4.91	20.15 ± 3.19	18.43 ± 4.16	19.20 ± 2.60
Lymphocytes (%)	73.88 ± 6.57	74.33 ± 4.48	75.15 ± 1.36	74.73 ± 3.80
MID (%)	6.98 ± 1.66	5.53 ± 1.64	6.43 ± 1.08	6.08 ± 1.41
VCM (fL)	54.03 ± 4.35	55.10 ± 3.99	53.03 ± 4.83	52.90 ± 3.96
MCH (pg)	20.03 ± 0.21	22.40 ± 3.80	20.13 ± 0.53	19.45 ± 0.10
Platelets (x10^3^/μL)	608.75 ± 72.38	686.50 ± 32.72	712.50 ± 85.44	737.75 ± 124.76
Females				
RBC (x10^6^/μL)	6.67 ± 0.84	6.46 ± 0.29	6.81 ± 0.40	6.88 ± 1.10
WBC (x10^3^/μL)	6.73 ± 1.20	6.90 ± 1.00	6.20 ± 1.24	6.58 ± 1.25
Hemoglobin (g/dL)	14.03 ± 1.35	13.73 ± 0.15	13.75 ± 0.96	14.23 ± 0.46
Hematocrit (%)	41.18 ± 4.07	40.48 ± 1.48	40.63 ± 2.51	41.60 ± 1.17
Neutrophils (%)	23.63 ± 3.09	22.83 ± 4.38	20.55 ± 4.01	22.30 ± 4.19
Lymphocytes (%)	69.68 ± 3.01	70.75 ± 4.99	73.00 ± 5.10	71.00 ± 4.69
MID (%)	6.70 ± 0.57	6.43 ± 0.79	6.45 ± 1.17	6.70 ± 1.44
MCV (fL)	55.98 ± 4.88	56.23 ± 3.01	55.53 ± 5.06	53.10 ± 5.20
MCH (pg)	21.58 ± 1.34	21.20 ± 0.97	20.90 ± 0.64	20.28 ± 0.35
Platelets (x10^3^/μL)	694.50 ± 115.36	734.25 ± 125.71	754.25 ± 48.13	612.50 ± 48.14

Data are presented as average ± standard deviation. There was no significant difference between the treated groups versus the control group. Tukey test after one-way analysis of variance. WBC, White Blood Cell; RBC, Red Blood Cell; MID, midrange absolute count (eosinophils, basophils, monocytes); MCH, medium corpuscular hemoglobin; MCV, medium corpuscular volume, MoAE=*M. oleifera* leaf aqueous extract

### Histopathological analysis

**Figure-1:**
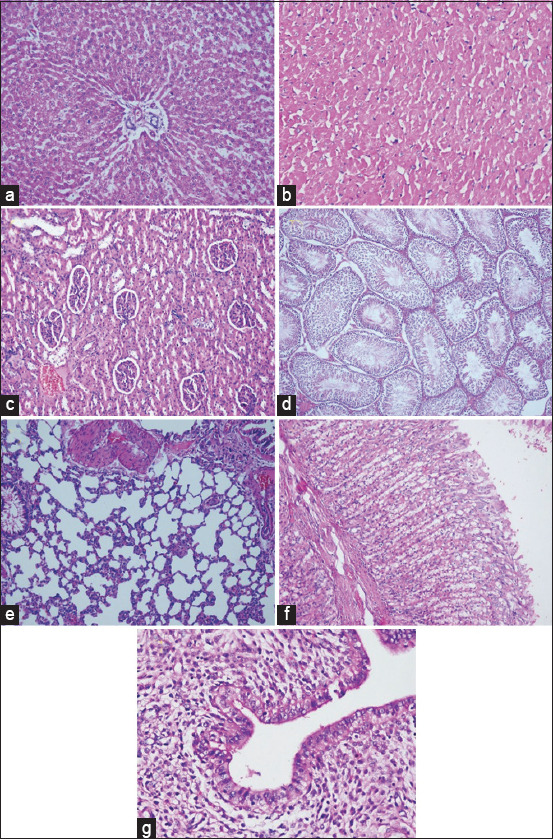
Microscopic images of organ tissues from rats subjected to a daily dose of 500 mg/kg of *Moringa oleifera* leaf aqueous extract for 28 days. No histopathological changes are evident in any of the organs. (a) Liver, (b) heart, (c) kidney, (d) seminiferous tubule, (e) lung, (f) stomach, and (g) uterus. Hematoxylin-eosin stain, 400×.

No alterations were observed in the histopathological examination of the liver, heart, kidney, stomach, lung, seminiferous tubules, and uterus of male and female rats treated orally with MoAE for 28 days at three dose levels (Figure-1).

### Effect on induced gout in rats

**Figure-2:**
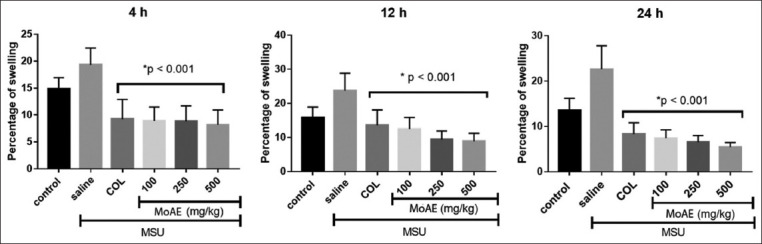
Effect of *Moringa oleifera* leaf aqueous extract on the percentage of swelling in arthritis induced in rats at 4, 12, and 24 h after monosodium urate (MSU) administration. COL denotes colchicine (0.30 mg/kg/day), while MSU represents monosodium urate (mg/mL). Values are presented as the arithmetic mean ± standard deviation. *p < 0.001 compared to the MSU group. The data were analyzed using a one-way analysis of variance followed by Tukey’s *post hoc* test.

The reduction in the percentage of ankle joint swelling in rats with crystal-induced MSU arthritis was notable after MoAE administration. An analogous result was observed in the colchicine-treated group. Evaluation performed 4 h after injection of MSU revealed a reduction in swelling percentages to 8.93 ± 2.57%, 8.82 ± 2.89%, and 8.17 ± 2.78% with doses of 100, 250, and 500 mg/kg/day, respectively, compared to 19.34 ± 3.10% in the MSU group (p < 0.001). The highest swelling percentage in the MSU group was observed 12 h after crystalloid injection, reaching 23.75 ± 5.09%. Treatment significantly decreased this value, with a more pronounced effect observed with the administration of MoAE at a dose of 500 mg/kg. A reduction in swelling percentage was confirmed at the 24-h mark, recording a value of 5.45 ± 1.02% with the administration of MoAE at a dose of 500 mg/kg (Figure-2).

**Figure-3:**
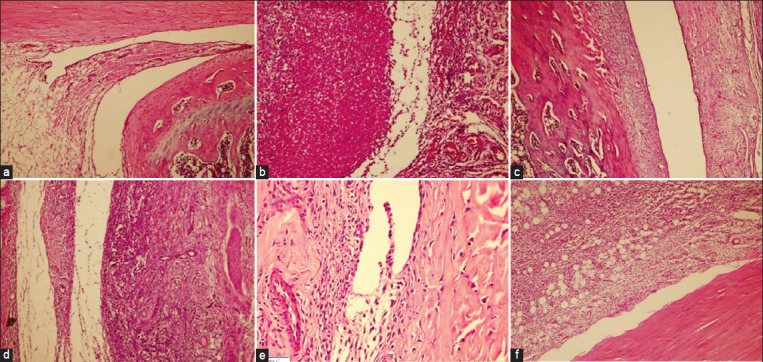
Photomicrographs showing ankle joints of rats with crystal-induced monosodium urate (MSU) arthritis. (a) Control group with normal conditions, (b) Group administered only with MSU, (c) Group treated with colchicine. (d-f) Groups treated with 100, 250, and 500 mg/kg of moringa, respectively.

The control group’s ankle joint histopathology showed an unobstructed joint space, healthy synovial membrane, and no inflammatory infiltrate (Figure-3a). Rats given only MSU crystal suspension exhibited a severe acute inflammatory infiltrate (Figure-3b). Figure-3c demonstrates a moderate presence of acute inflammatory infiltrate in the colchicine-treated group. At doses of 100 and 250 mg/kg, groups receiving MoAE exhibited moderate inflammatory infiltrate (Figures-3d and e), while at 500 mg/kg, the inflammatory infiltrate was mildly present (Figure-3f).

### Effect on hyperuricemia

**Figure-4:**
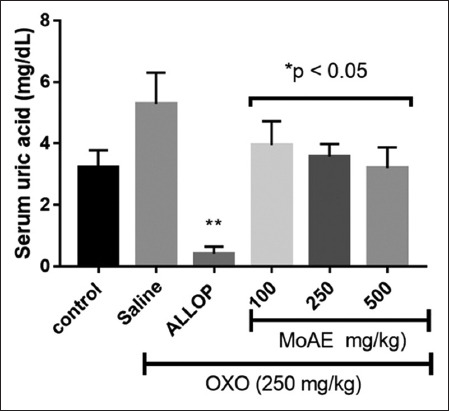
Serum uric acid concentrations in rats experiencing oxonate-induced hyperuricemia (OXO) and subjected to *Moringa oleifera* leaf aqueous extract treatment. ALLOP represents allopurinol (50 mg/kg). Data were presented as mean ± standard deviation. *p < 0.05 and **p < 0.001 compared to the oxonate group (saline).

Oxonate administration elevated uric acid levels in the serum of rats, with a more pronounced impact observed in the group treated solely with saline solution (oxonate group). Treatment with MoAE exhibited a significant reduction in these values in a dose-dependent manner. At a dose of 100 mg/kg/day, a decrease was observed at 3.95 ± 0.77 mg/dL (a reduction of 25.33%), and at 500 mg/kg/day, a decrease was observed at 3.20 ± 0.66 mg/dL (a reduction of 39.51%) compared to 5.29 ± 1.01 mg/dL of the oxonate group (p < 0.05). The outcome observed with 500 mg/kg closely resembled 3.23 ± 0.55 mg/dL in the normal control group (Figure-4).

### Analgesic effect

**Figure-5:**
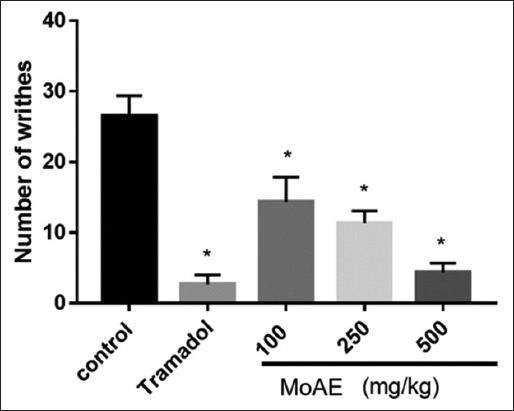
Effect of *Moringa oleifera* leaf aqueous extract on acetic acid-induced writhes. Values are presented as mean ± standard deviation. *p < 0.001 compared to the control group.

The analgesic effect of MoAE was evident in a dose-dependent manner. At 100 mg/kg/day, the observed number of writhes was 14.33 ± 3.50, compared to 26.5 ± 2.88 in the control group, indicating a 45.92% inhibition (p < 0.001). With a dose of 500 mg/kg, there was a reduction in writhes to 4.33 ± 1.37, representing an inhibition of 83.66%, a value closely aligned with the 89.92% inhibition produced by tramadol (Figure-5).

## Discussion

At the maximum dose of 2000 mg/kg for an acute oral toxicity test and in the 28-day study with repeated doses, MoAE showed no toxic effects. Table-3 indicates no reported rat deaths and no observed changes in liver, kidney, or lipid profile functions. In Table 4 and Figure 1, no abnormalities in blood tissue or organs were detected. In Table-2, body weight gain did not change.

The results reported by Adedapo *et al*. [25] and Awodele *et al*. [26] align with our findings. No acute or subacute toxicity was detected at doses up to 2000 mg/kg. 2000 mg/kg infusion of *M. oleifera* powder did not elicit changes in biochemical, hematological, histological levels or weight gain. The infusion administration did not reveal any subchronic toxicity. 500 and 1000 mg/kg of the powder caused liver and renal toxicity [27]. A dose of 3000 mg/kg, but not 1000 mg/kg, of *M. oleifera* aqueous leaf extract caused genotoxicity in human mononuclear blood cells [28].

The results of research on diverse moringa extracts varied due to differing chemical compositions caused by distinct secondary metabolite extractions depending on their polarity. Eight-week administration of *M. oleifera* leaves’ methanolic extract led to liver and kidney toxicity [29]. No rat deaths were reported during the acute toxicity test with *M. oleifera*’s methanolic extract. During the 90-day subchronic toxicity study with the dose of 600 mg/kg, both body weight loss and reductions in LDL and cholesterol occurred. Histopathological examination failed to reveal any structural changes [30]. 1000 mg/kg dosage of *M. oleifera* leaf hydroethanolic extract caused moderate hepato-nephrotoxicity in mice after 28 days. At lower doses, it was deemed safe [31].

The study’s findings suggest that this plant can be safely used in alternative and complementary medicine. While no toxic effects were noted, *M. oleifera* leaf extract has been shown to provide protection against certain toxicities. It has been reported to protect against tilmicosin-induced renal damage [32], levofloxacin-induced liver toxicity [33], and streptozotocin-induced liver toxicity in rats [34]. The study revealed protection against *Echis ocellatus* snake venom toxicity through antioxidant and anti-inflammatory mechanisms [35]. It protects against cardiac damage caused by aluminum phosphide intoxication [36]. At the testicular level, the extract has shown protective effects against the toxic effects of tramadol [37], neurotoxicity caused by rotenone altering the function of the testicular gonadal axis [38], toxic effects of highly active antiretroviral therapy [39], and cyclophosphamide-induced testicular toxicity [40]. It alleviated the harmful effects of sodium arsenate on mouse embryos [41].

Table-1 shows the presence of phenolic compounds, such as flavonoids in the MoAE. The study by Manguro and Lemmen [42] found rutin and quercetin flavonoids in *M. oleifera*’s phenolic compounds. The antioxidant capacity of MoAE was measured at 7.91 ± 0.10 μg ascorbic acid equivalent/mg. Aa *et al*. [43] have reported the antioxidant activity of *M. oleifer*a leaf extract while other researchers [44, 45] reported the antioxidant activity of *M. oleifer*a phenolic compounds. Phenolic compounds exhibit antioxidant properties by interacting with free radicals [46].

In the investigation of MSU-induced gouty arthritis, MoAE showed a significant reduction in the percentage of ankle joint swelling in rats, as illustrated in Figure-1. Furthermore, there was a noticeable decrease in inflammatory infiltrate within the joints, evident at the histopathological level, as shown in Figure-2. These findings suggest an anti-inflammatory and antiarthritic effect of MoAE. This is consistent with Fatima and Fatima in 2016 [8], which demonstrated substantial antiarthritic activity in rats with formaldehyde-induced arthritis in the subplantar region. Furthermore, Mittal *et al*. [6] showcased the anti-inflammatory effect in rats using 200 mg/kg doses of MoAE in models induced by carrageenan and formaldehyde paw edema, as well as cotton pellet-induced granuloma. Tsala *et al*. [7] reported that the hot water extract of *M. oleifera* leaves exerted an anti-inflammatory effect on ear edema, paw edema, and cotton pellet-induced granuloma. The study’s findings of antiarthritic and anti-inflammatory effects can be partly explained by the flavonoids in MoAE (Table-1). Flavonoids have demonstrated antiarthritic activity primarily by suppressing inflammation-triggering cytokines such as IL-6 [47] and inhibiting Th17 cells that produce IL-17 [48].

The decrease in persistent hyperuricemia is important because uric acid crystals are deposited in intraarticular and periarticular spaces and activate the innate immune system, generating the most common arthritis in men, gouty arthritis [49]; the decrease in hyperuricemia would enhance gouty arthritis’ anti-inflammatory effects. Figure-3 depicts a dose-dependent decrease in uricemia levels resulting from MoAE treatment. The inhibition of xanthine oxidase activity and uric acid production by *M. oleifera*’s leaf hydrolysate fractions, as found by Tian *et al*. [50], corroborates this result. Flavonoids such as quercetin, apigenin, and scutellarein, as well as tannins, stand out as promising new xanthine oxidase inhibitors, as suggested by recent findings [51].

Since gout is a progressive, painful, and debilitating form of inflammatory arthritis, we assessed MoAE’s analgesic effect. At a dose of 500 mg/kg, the drug showed an analgesic effect comparable to tramadol, as shown in Figure-5. Consuming MoAE, rich in flavonoids (Table-1), may help alleviate gouty arthritis symptoms. Flavonoids exhibit pain-relieving properties [52]. Quercetin, a flavonoid, exhibits antinociceptive effects in murine models of inflammatory, oncologic, and neuropathic pain. The opioidergic system is modulated, and oxidative stress is suppressed through the mechanism of action [53].

## Conclusion

In experimental tests, *M. oleifera* leaf extract showed no acute or subacute oral toxicity. In a murine model, it exhibited antiarthritic, antihyperuricemic, and analgesic effects. The phenolic content and antioxidant activity of these substances may account for their observed effects.

Limitations include that the study was conducted in rats and that the results may not be directly applicable to humans due to physiological and metabolic differences between species. Furthermore, the study did not investigate the mechanisms underlying the observed effects, which limits our understanding of how MoAE acts at the molecular or cellular level. Regarding the future scope of the study, it is crucial to conduct long-term investigations to assess chronic toxicity and possible prolonged side effects. It is also important to explore the biochemical and molecular mechanisms of the antiarthritic, antihyperuricemic, and analgesic effects of MoAE, as well as to study the absorption, distribution, metabolism, and excretion (ADME) of MoAE to better understand its pharmacokinetic and pharmacodynamic properties. In addition, the development and testing of different formulations of MoAE may improve its bioavailability, stability, and patient acceptance.

## Authors’ Contributions

JPRA and MPP: Study conception and design. MPP and JTMH: Collected the plant species. JLAA: Carried out the preparation of extract. JPRA, MPP, and HJJG: Performed the experiments. JMOS: Histopathological analysis. JTMH: Statistical analysis. JPRA and JLAA: Drafted and revised the manuscript. All authors have read, reviewed, and approved the final manuscript.
